# Molecular Epidemiology, Antimicrobial Surveillance, and PK/PD Analysis to Guide the Treatment of *Neisseria gonorrhoeae* Infections

**DOI:** 10.3390/pharmaceutics13101699

**Published:** 2021-10-15

**Authors:** Rodrigo Alonso, Ainara Rodríguez-Achaerandio, Amaia Aguirre-Quiñonero, Aitor Artetxe, Ilargi Martínez-Ballesteros, Alicia Rodríguez-Gascón, Javier Garaizar, Andrés Canut

**Affiliations:** 1 Department of Immunology, Microbiology and Parasitology, Faculty of Pharmacy, University of the Basque Country UPV/EHU, 01006 Vitoria-Gasteiz, Spain; rodrigo.alonso@ehu.eus (R.A.); a.artetxe.arrate@gmail.com (A.A.); ilargi.martinez@ehu.eus (I.M.-B.); javier.garaizar@ehu.eus (J.G.); 2 Bioaraba Microbiology, Infectious Disease, Antimicrobial Agents, and Gene Therapy Group, 01009 Vitoria-Gasteiz, Spain; ainara.rodriguezachaerandio@osakidetza.eus (A.R.-A.); amaia.aguirrequinonero@osakidetza.eus (A.A.-Q.); 3 Microbiology Service, Araba University Hospital, Osakidetza Basque Health Service, 01009 Vitoria-Gasteiz, Spain; 4 Pharmacokinetic, Nanotechnology and Gene Therapy Group (PharmaNanoGene), Faculty of Pharmacy, University of the Basque Country UPV/EHU, 01006 Vitoria-Gasteiz, Spain

**Keywords:** *N. gonorrhoeae*, antibiotic resistance, cephalosporins, azithromycin, pharmacokinetic/pharmacodynamic (PK/PD) analysis, whole-genome sequencing (WGS)

## Abstract

The aim of this study was to apply molecular epidemiology, antimicrobial surveillance, and PK/PD analysis to guide the antimicrobial treatment of gonococci infections in a region of the north of Spain. Antibiotic susceptibility testing was performed on all isolates (2017 to 2019, *n* = 202). A subset of 35 isolates intermediate or resistant to at least two antimicrobials were selected to search for resistance genes and genotyping through WGS. By Monte Carlo simulation, we estimated the probability of target attainment (PTA) and the cumulative fraction of response (CFR) of the antimicrobials used to treat gonorrhea, both indicative of the probability of treatment success. In total, 2.0%, 6.4%, 5.4%, and 48.2% of the isolates were resistant to ceftriaxone, cefixime, azithromycin, and ciprofloxacin, respectively. Twenty sequence types were identified. Detected mutations were related to antibiotic resistance. PK/PD analysis showed high probability of treatment success of the cephalosporins. In conclusion, multiple populations of *N. gonorrhoeae* were identified. We can confirm that ceftriaxone (even at the lowest dose: 250 mg) and oral cefixime are good candidates to treat gonorrhea. For patients allergic to cephalosporins, ciprofloxacin should be only used if the MIC is known and ≤0.125 mg/L; this antimicrobial is not recommended for empirical treatment.

## 1. Introduction

Sexually transmitted infections (STIs) are a serious global health problem [1]. The prevalence of gonorrhea has increased significantly in recent years. The World Health Organization (WHO) estimates that there are 87 million new cases of gonorrhea each year [2].

In recent years, the most frequently prescribed antibiotics for the treatment of gonorrhea have become less effective, in part due to the inexorable progression of gonococcal antimicrobial resistance [3,4]. In 2018, the WHO reported the expansion of multidrug-resistant strains of *N. gonorrhoeae* worldwide [5]. In Europe, the Euro-GASP (depending on the European Centre for Disease Prevention and Control) provides important data at the European level on antimicrobial resistance, which are used to inform treatment guidelines [6]. Thus, the determination of antimicrobial susceptibility to identify isolates with less susceptibility and resistance to antimicrobial agents, and monitoring of strain populations via molecular techniques to identify gonococcal clones that are important for driving the transmission of multidrug-resistant gonococci, become crucial. Additionally, ensuring effective empirical therapy is also essential.

Current guidelines—such as the European guidelines for the diagnosis and treatment of gonorrhea in adults [7], and the Center for Disease Control and Prevention (CDC) Treatment Guidelines for Gonococcal Infection [8], both from 2020—recommend a dual therapy with extended-spectrum cephalosporins (ESCs, such as ceftriaxone or cefixime) and azithromycin for the treatment of uncomplicated gonorrhea when the antimicrobial susceptibility is unknown. Some guidelines recommend fluoroquinolones as an alternative treatment of pharyngeal infections if the isolate is known to be fluoroquinolone-susceptible and there are indications against using ceftriaxone—for instance, history of severe hypersensitivity to cephalosporins [7,9].

Recently, improved methods for the evaluation of the effects of pharmacokinetics (PK) and pharmacodynamics (PD) on treatment outcomes have become available for a number of other infections. In this regard, better data and new research on PK contributors to gonorrhea treatment outcomes are needed [10]. PK/PD analysis integrates information about the concentration of the drug that reaches the infection site and induces the therapeutic response, and the susceptibility of the pathogen to the antibiotic, expressed as the minimum inhibitory concentration (MIC). This allows researchers or clinicians to select the optimal antibiotic and dosing regimen for each infectious process and patient in order to enhance the effect of the antibiotic, minimizing the incidence of side effects and the emergence of resistance [11].

The aim of this work was to apply molecular epidemiology—including whole-genome sequencing (WGS) information, antimicrobial surveillance data, and PK/PD analysis—to guide the antimicrobial treatment of gonococci infections.

## 2. Materials and Methods

### 2.1. N. gonorrhoeae Isolates

All isolates from 2017 to 2019 were collected at the Microbiology Service of the University Hospital of Araba (HUA). The hospital, located in the Basque Country (Spain), covers a population of ~400,000 inhabitants, and has 780 beds. 

Non-duplicated *N. gonorrhoeae* isolates from patients attending to the Emergency, Infectious Disease, and Primary Care services were included in the study. Gonococcal isolation was performed on chocolate agar PolyViteX VCA and Chocolate agar PVX plates (bioMérieux, Marcy-l’Étoile, France). The suspected colonies were then identified by mass spectrometry using the MALDI-TOF MS (Microflex LT, Bruker-Daltonics, Bremen, Germany) methodology [12]. 

This study met the exemption criteria of the ethics committee of clinical research because the isolates analyzed were collected in routine practice, and did not allow the identification of patients.

### 2.2. Antibiotic Susceptibility Testing

Isolated strains were subjected to antibiotic susceptibility testing using the MIC gradient strip tests (Liofilchem, Roseto degli Abruzzi, Italy) according to the manufacturer’s instructions. The minimum inhibitory concentrations (MICs) of tetracycline, penicillin, cefixime, ceftriaxone, ciprofloxacin, and azithromycin were determined. From the MIC distribution data of all of the isolates, the calculation of MIC50 and MIC90—i.e., the lowest concentration of the antimicrobial capable of inhibiting 50% and 90% of the isolates, respectively—was performed. Resistance rates were calculated with reference to the European Committee on Antimicrobial Susceptibility Testing (EUCAST) [13] and the Clinical and Laboratory Standards Institute (CLSI) [14]. The current EUCAST MIC breakpoints for penicillin are ≤0.06 mg/L (susceptible) and >1 mg/L (resistant); for ceftriaxone and cefixime, ≤0.125 mg/L (susceptible) and >0.125 mg/L (resistant); for ciprofloxacin, ≤0.03 mg/L (susceptible) and >0.06 mg/L (resistant); and for tetracycline, ≤0.5 mg/L (susceptible) and >1 mg/L (resistant). For azithromycin, the EUCAST epidemiological cutoff (ECOFF) of 1 mg/L was used to identify non-wild-type (WT) isolates. CLSI breakpoints were the following: for penicillin, ≤0.06 mg/L (susceptible), 0.12–1 mg/L (intermediate), and ≥2 mg/L (resistant); for ceftriaxone and cefixime, ≤0.25 mg/L (susceptible); for azithromycin, ≤1 mg/L (susceptible); for ciprofloxacin, ≤0.06 mg/L (susceptible), 0.12–0.5 mg/L (intermediate), and ≥1 mg/L (resistant); and for tetracycline, ≤0.25 mg/L (susceptible), 0.5–1 mg/L (intermediate), and ≥2 mg/L (resistant).

### 2.3. WGS Molecular Epidemiology and Antimicrobial Resistance Determinants

A subset of 35 strains intermediate or resistant to at least two of the antimicrobials tested were selected to search for resistance genes and genotyping through WGS. The DNA of the strains was extracted using the NucleoSpin Tissue Kit (Macherey-Nagel, Duren, Germany) and sequenced using the Illumina MiSeq platform with the Nextera DNA Flex Library Prep Kit (Illumina, San Diego, CA, USA). The genomic information was received as small nucleotide sequence reads, and longer sequences as contigs. These sequence data were submitted to GenBank under the BioProject accession number PRJNA684048 (https://www.ncbi.nlm.nih.gov/sra/PRJNA684048, accessed on 10 December 2020). Subsequently, the assembled genomes of the isolates were input to the ResFinder 3.2 tool on the CGE website (https://cge.cbs.dtu.dk, accessed on 10 December 2020) for the identification of acquired antimicrobial resistance genes and chromosomal mutations. Default thresholds of 90% identity and 60% gene coverage were employed. Additionally, the PubMLST tool was employed to detect antimicrobial resistance determinants (https://pubmlst.org/neisseria/, accessed on 10 December 2020). Multilocus sequence typing (MLST) and *N. gonorrhoeae* multi-antigen sequence typing (NG-MAST) were performed on the sequences produced by WGS, and they were compared with existing alleles on the Neisseria MLST website (http://pubmlst.org/neisseria/, accessed on 10 December 2020) and NG-MAST (http://www.ng-mast.net/, accessed on 10 December 2020) schemes for the determination of allele numbers and sequence types (STs). All new alleles or STs were submitted to the NG-MAST or MLST website curators to be assigned an allelic number and ST. The genomic epidemiology of the isolates was determined based on whole-genome analysis. An SNP phylogenetic tree, based on FASTQ files, was generated by using the default settings of the MINTyper 1.0 server, available at https://cge.cbs.dtu.dk/services/MINTyper/ (accessed on 10 December 2020). The complete genome of isolate FA1090 (NC_002946.2) was used as a reference. Furthermore, a genome-based clustering was also performed using the TYGS platform, accessible at https://tygs.dsmz.de/ (accessed on 10 December 2020). 

### 2.4. Pharmacokinetic/Pharmacodynamic (PK/PD) Analysis

In order to predict the probability of PK/PD target attainment, different antibiotics and dosing regimens were evaluated (Table 1). 

From the published pharmacokinetic parameters listed in Table 1 [15,16,17,18,19], and from the study sample MIC distribution, we estimated the probability of target attainment (PTA, defined as the probability that at least a specific value of a PK/PD index is achieved at a certain minimum inhibitory concentration) and calculated the cumulative fraction of response (CFR, defined as the expected population probability of target attainment for a specific drug dose and a specific population of microorganisms). PK/PD indices and values related to efficacy are also listed in Table 1. Five-thousand-subject Monte Carlo simulations with Oracle^®^ Crystal Ball Fusion Edition v.11.1.2.3.500 (Oracle USA Inc., Redwood City, CA, USA) were used to estimate the PTA and CRF values, indicative of the probability of target success. PTA and CFR ≥ 80% but < 90% were associated with moderate probabilities of success, whereas a CFR ≥ 90% was considered optimal against that bacterial population. More detailed information about the PTA and CFR calculations is presented in the Supplementary Materials.

## 3. Results

### 3.1. N. gonorrhoeae Isolates and Antibiotic Susceptibility Testing

A total of 202 isolates of *N. gonorrhoeae* were collected in the study period (2017–2019). Of these, 85% were collected from men, and 15% from women. In men, the urethral exudate was the most common sample (87%); other locations were anal exudate (11%) and pharyngeal exudate (2%). Endocervical (64%) and vaginal exudate (33%) were the most common among women, and anal exudate (1%) was also detected. The average age was 31 years (range 14–67) and 33 years (age range 16–61) for men and women, respectively. A total of 10 patients had *N. gonorrhoeae*-positive cultures in two anatomical sites (7 pharynx and rectum, 3 pharynx and urethra), but only one isolate per patient was included in the susceptibility study. 

Table 2 shows the antimicrobial susceptibility of *N. gonorrhoeae* isolates. The higher resistance rates were obtained for ciprofloxacin and tetracycline. Eleven isolates were resistant to azithromycin (5.4% of the total), all of which were collected in 2019. Resistant isolates had MICs of 1.5 mg/L (*n* = 5), 2 mg/L (*n* = 5), and 16 mg/L (*n* = 1). The percentage of isolates resistant to cephalosporins varied depending on the EUCAST and CLSI clinical breakpoints: for cefixime, resistance rates were 6.4% (EUCAST) and 1.6% (CLSI), and for ceftriaxone they were 2.0% (EUCAST) and 0.5% (CLSI). The MIC distribution of the six antibiotics is presented in Figure S1.

### 3.2. Genotyping: MLST and NG-MAST

MLST genotyping identified multiple distinct populations of *N. gonorrhoeae* in the isolates of HUA in 2017–2019 (Table 3). Overall, a total of 20 MLST STs were identified among the 35 isolates analyzed, with ST-9363 being the most common (*n* = 8), followed by ST-7363 (*n* = 4) and ST-7822 (*n* = 3), while 14 STs were represented by a single isolate. Two new MLST STs (ST-14274 and ST-14304) were previously unreported, and resulted from combinations of known alleles. NG-MAST further discriminated between strains with identical MLST-STs, and 27 different NG-MAST STs were observed among the 35 isolates analyzed. ST-6765 predominated (*n* = 7), ST-470 and ST-13070 were observed in two isolates, and the remaining STs were represented by single isolates. Additionally, six NG-MAST STs (17.1%) were previously unreported, and resulted from new combinations of known alleles. 

### 3.3. Antimicrobial Resistance Determinants

Genomic markers associated with resistance to ciprofloxacin, tetracycline, β-lactams, and azithromycin were found (Table 3). All of the ciprofloxacin-resistant strains investigated presented two substitutions in GyrA and one or two substitutions in ParC. At GyrA, they presented substitutions in positions 91 (all presented an S91F) and 95 (D95A/G/N). The main amino acid substitution observed in ParC was S87R (*n* = 13), either alone, or associated with S88P. Other substitutions included D86N and E91G. 

The *tetM* gene was detected in the four highly tetracycline-resistant strains (MIC ≥ 24 mg/L). The V57M substitution in the ribosomal protein S10 subunit encoded by the *rpsJ* gene was detected in 33/35 (94.3%) of the strains investigated, irrespective of their tetracycline susceptibility. Chromosomal mutations in the *porB* gene were also found in tetracycline-resistant strains. The G120K substitution was detected in 14 isolates, and was associated with A121G/N/D. 

The only sequenced isolate resistant to ceftriaxone (MIC: 0.25 mg/L) presented the *penA* mosaic allele XXXIV associated with an L421P substitution in PBP1, a G120K/A121N in the PorB, and an adenine deletion (-35A Del) in the *mtrR* promoter.

Twenty-five isolates showed different degrees of penicillin resistance. The four high-level penicillin-resistant strains (*n* = 4; MIC ≥ 2 mg/L) carried the *bla*_TEM-1B_ gene, which encodes the TEM-1 β-lactamase; these four strains were β-lactamase producers, as determined by the nitrocefin test. The remaining isolates (*n* = 21) presented an MIC range between 0.19 and 0.75 mg/L. Nineteen isolates were associated with non-mosaic *penA* alleles, with non-mosaic II being the most common (*n* = 10); the remaining six isolates were associated with mosaic *penA* alleles (mosaic X and XXXIV, *n* = 3 each). The major amino acid substitution observed in the PBP1 protein was Leu421Pro (*n* = 13), which was correlated with a degree of intermediate resistance. Simultaneous substitutions at amino acids G120 and A121 in the PorB porin—associated with decreased intake of several antimicrobials—were observed in 16 penicillin-resistant isolates, with the substitutions of G120K/A121N being the most common (*n* = 10). 

The 13 isolates with an MIC of 1–2 mg/L against azithromycin were associated with mutations in the promoter region and coding sequence of the *mtrR* genes, and with mutations in the gene encoding the 23S rRNA. Two isolates presented the C2599T mutation in the 23S rRNA gene associated with a G45D substitution in the MtrR protein. A deletion of one guanine (G), 20 nucleotides downstream of the -35A deletion, was observed in the *mtrR* promoter of 11 isolates; their sequences showed a 98.48% identity with that of strain 38,194 (GenBank accession number KT954125), and 86.36% with strain FA1090 (GenBank accession number NC_002946). The -35A deletion in the repeated sequence of the *mtrR* promoter was not observed in the resistant isolates; however, it was observed in six susceptible isolates (MIC, <0.5 mg/L) as the only mechanism related to azithromycin resistance. In the MtrR protein, the amino acid substitutions of A39T (*n* = 3) and G45D (*n* = 2) were detected in azithromycin-resistant strains. The alignment of partial sequences is presented in Supplementary Figure S2.

**Figure 1:**
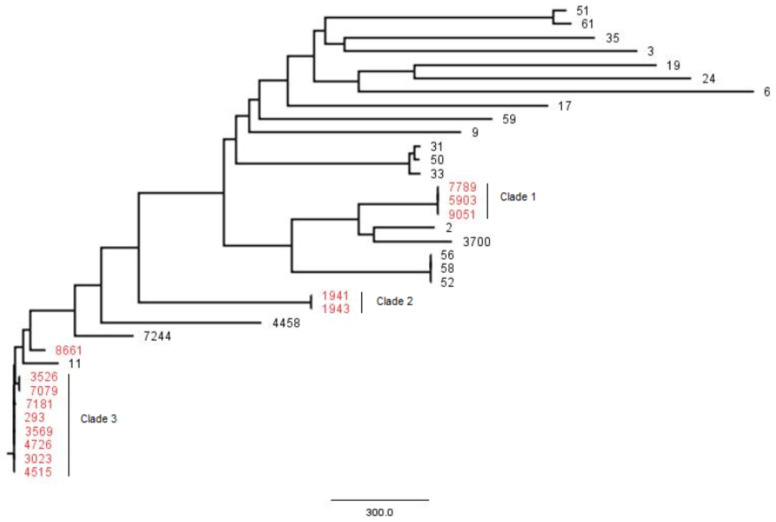
Clustering of the 35 *N. gonorrhoeae* sequences obtained by WGS, based on genome SNPs; red indicates non-azithromycin-susceptible isolates.

Whole-genome SNP-based phylogenetic analysis of the 35 sequenced isolates revealed a great genetic dissimilarity between isolates, with differences ranging from 0 to 2378 SNPs; however, three clades involving non-azithromycin-susceptible isolates were identified (Figure 1). Clade 1 consisted of three isolates belonging to MLST ST-7822, with an Ala39Thr substitution in the *MtrR* gene and a deletion of one G, 20 nucleotides downstream of where the -35A deletion occurs; no SNP differences were observed. Clade 2 consisted of two isolates belonging to MLST ST-1580, with the C2599T mutation in the *23S rRNA* gene and a Gly45Thr substitution in the *MtrR* gene; no SNP differences were observed. Clade 3 consisted of eight closely related isolates belonging to MLST ST-9363, with a deletion of one G, 20 nucleotides downstream of where the -35A deletion occurs; differences between these isolates ranged from 0 to 20 SNPs. The same three clusters were obtained when the phylogenetic tree was constructed from the whole genome (data not shown).

### 3.4. Pharmacokinetic/Pharmacodynamic (PK/PD) Analysis

**Figure 2:**
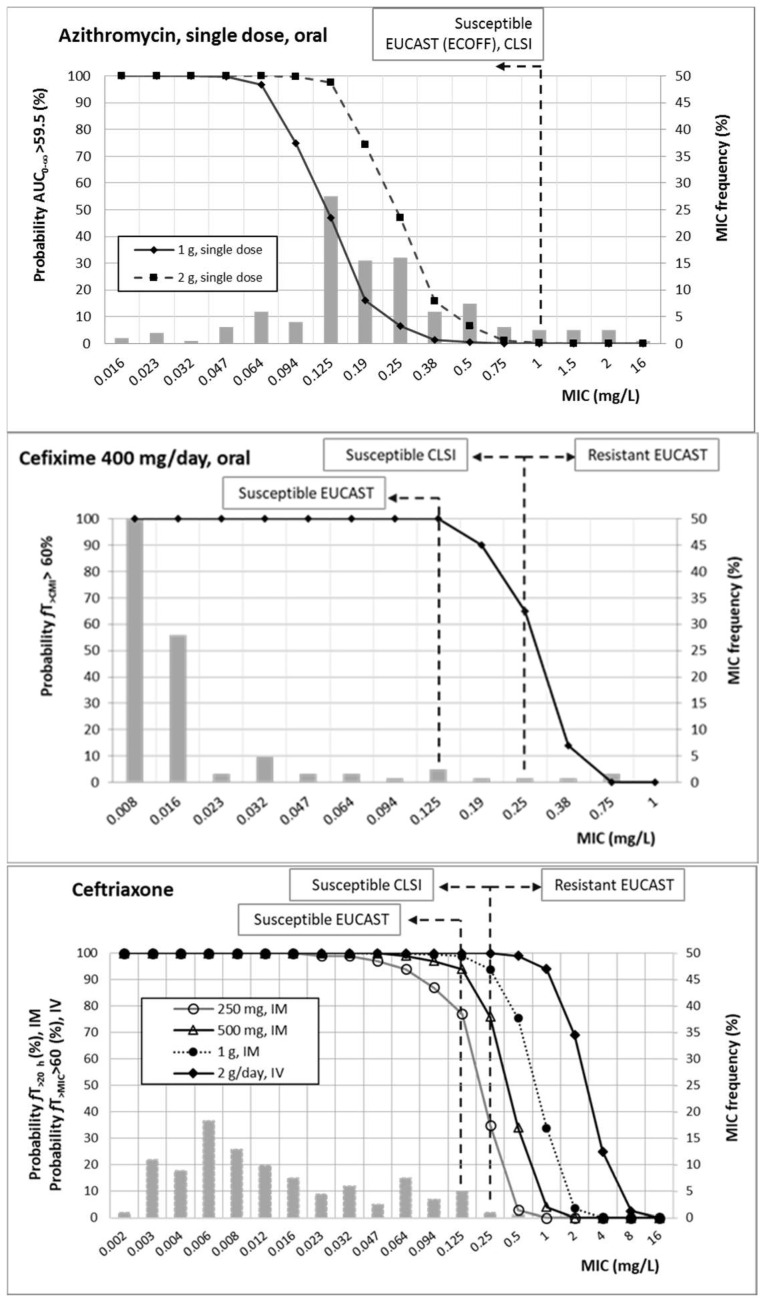

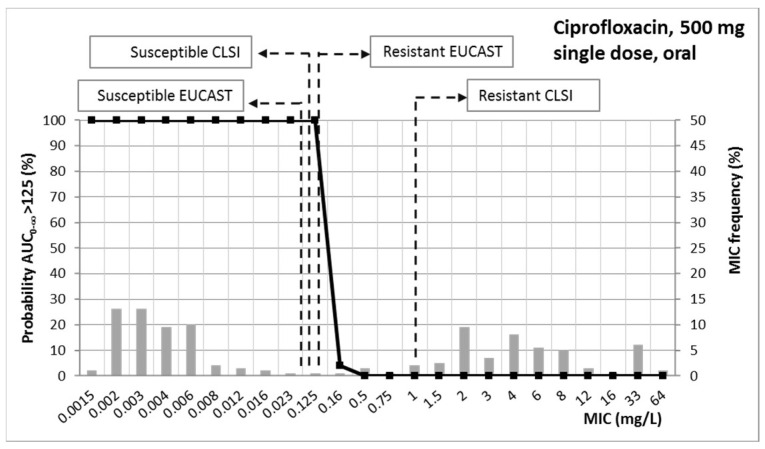
Probability of target attainment (PTA) of azithromycin, ceftriaxone, cefixime, and ciprofloxacin. Grey bars: MIC frequency.

Figure 2 shows the probability of target attainment (PTA) values of all of the antimicrobial agents studied, as well as their MIC distribution. On the basis of the simulation results, and taking into account the targets for the different antimicrobial agents, PTA values equal to or higher than 90% were achieved with (1) azithromycin at single doses of 1 g and 2 g, for MICs up to 0.064 and 0.125 mg/L, respectively; (2) ceftriaxone at 250, 500, and 1000 g, administered intramuscularly as single doses, for MICs up to 0.064, 0.125, and 0.25 mg/L, respectively, and for MICs equal to or lower than 1 mg/L for 2 g/day via the intravenous route; (3) cefixime at 400 mg/day by oral route, for MICs up to 0.125 mg/L; and (4) single oral doses of ciprofloxacin at 500 mg, for MICs up to 0.125 mg/L. 

Table 4 features the CFR values obtained after the PK/PD analysis and Monte Carlo simulations. Values higher than 90% were only achieved with the two cephalosporins. 

In supplementary Tables S2–S9, the mean, median, and 2.5% and 97.5% confidence intervals of PTA and CFR for every antimicrobial and dosing regimen are presented.

## 4. Discussion

To address the antimicrobial resistance problem, more efficient means of evaluating therapies for gonorrhea are needed. Our purpose in this study was to apply molecular epidemiology—including whole-genome sequencing (WGS) information, antimicrobial surveillance data, and PK/PD analysis—to guide the antimicrobial treatment of gonococci infections in the Basque Country (Spain). 

In this region of the north of Spain, the percentage of resistance was of the same order as recent data obtained from Madrid [20] and Barcelona [21,22], and also of those reported in other European countries [23]. As expected, the highest percentage of resistance was detected for ciprofloxacin (>45%). The historical resistance of gonococci to ciprofloxacin is very well known, and it has been related to the incidence of gonorrhea at the population level. In fact, ciprofloxacin is no longer recommended for treatment of this STI, except for pharyngeal infections if susceptibility has been proven or if the patient is allergic to β-lactams [9].

In spite of the differences in the resistance breakpoint between EUCAST [13] and CLSI [14] for ciprofloxacin (0.125 and 1 mg/L, respectively), the percentage resistance we obtained was similar, since very few isolates are affected by the discrepancies in the breakpoints (Figure 1). However, discrepancies in the clinical breakpoint for cephalosporins (0.25 mg/L from EUCAST and 0.5 mg/L from CLSI) justify the difference in the percentage resistance for cefixime (6.5% vs. 1.6%) and ceftriaxone (2.0% vs. 0.5%). Unlike the other antibiotics, all isolates resistant to azithromycin were collected in 2019 (*n* = 11). These data are consistent with the recent increase in the resistance of *N. gonorrhoeae* to azithromycin in Spain and worldwide [22,23]. Some studies support the notion that the selection/induction of azithromycin resistance in *N. gonorrhoeae* may be associated with the general use of azithromycin for the treatment of respiratory infections or non-gonococcal urethritis [24]. According to the susceptibility profile, azithromycin and cephalosporins are the most active antimicrobials. These results are in agreement with the European guidelines, which recommend the use of a dual therapy with ceftriaxone and azithromycin for all gonorrhea cases [7]. 

Antibiotic susceptibility data were consistent with those obtained through WGS, and the mutations detected were related to resistance to the studied antibiotics. The genomic study detected the main antimicrobial resistance determinants associated with resistance to β-lactams (*penA*, *porB*, *ponA*, *mtrR*, and *bla*_TEM1-B_), ciprofloxacin (*gyrA* and *parC*), and tetracycline (*tetM* and *rpsJ*). All ciprofloxacin-resistant strains presented two substitutions in GyrA, together with substitutions in the ParC protein, while among the ciprofloxacin-susceptible isolates, no mutations in the *gyrA* and *parC* genes were detected, as previously reported [25]. Regarding ceftriaxone—and as expected—the mutations found in the analyzed resistant isolates were associated with reduced susceptibility or resistance to ESC [26]. Tetracycline resistance in *N. gonorrhoeae* may be related to the presence of the plasmid-mediated TetM protein (MIC, 16–64 mg/L), or to mutations in some chromosomal genes (MIC, 2–4 mg/L) [27,28]. In our study, the *tetM* gene was detected only in high-level resistant isolates; meanwhile, low-level resistant isolates only presented mutations in the chromosomal genes *penB* (PorB), *rpsJ* (S10), and *mtrR* and its promoter. These isolates presented at least two or three mutated genes, suggesting that more than one antimicrobial resistance determinant is needed to confer resistance.

Resistance to β-lactams, macrolides, and tetracycline may result from overexpression of the MtrCDE efflux pump related to mutations in the promoter and/or coding regions of the *mtrR* repressor, which causes not only resistance, but also an increase in the MIC [25]. The -35A deletion in the promoter sequence—the most common mutation—was detected only in isolates recovered in 2017 and 2018, but it was not observed in those from 2019. Several mutations—including insertions, deletions, or transversions—have been described in this promoter region [29]. Related to azithromycin resistance, a guanine (G) deletion in this promoter was observed in most of the azithromycin-resistant isolates, either alone or associated with the A39T substitution in MtrR repressor. In some isolates, this mutation was associated with substitutions in the MtrD protein (data not shown), so the exact contribution of this G deletion to antimicrobial resistance should be determined.

The majority of genotyped azithromycin-resistant isolates were MLST ST-9363. In a recent study carried out in Barcelona (Spain) [22], this genotype was associated with outbreaks of *N. gonorrhoeae* with high-level azithromycin resistance (MIC ≥ 256 mg/L) between 2016 and 2018. In that study, this genotype was found mainly in men who have sex with men. Contrary to Barcelona isolates, our ST-9363 isolates were not high-level azithromycin resistant, and their MIC values were 1 and 2 mg/L. The phylogenetic analysis of both the SNPs and the complete genome using the WGS showed similar results and, together with the application of the other epidemiological markers and antibiotic resistance, allowed the epidemiological characterization of the strains analyzed. The joint analysis of the data obtained from the WGS allowed the detection of three clusters that involved eight, three, and two non-azithromycin-susceptible isolates, respectively, all of them isolated in 2019 and with an MIC ≥ 1 mg/L to azithromycin.

Among the interventions to mitigate the current and future impact of antimicrobial resistance, the development of new generations of antimicrobials is one of the most accepted. Another recognized strategy to diminish antibiotic resistance is the optimization of the dosing regimen of available antimicrobials. PK/PD analysis and Monte Carlo simulation have shown to be very useful tools to select adequate antibiotic dosages, with the goal of increasing treatment efficacy and reducing the risk of multidrug-resistant pathogens [30]. Although integrated PK/PD analysis has been applied to identify changes in the antimicrobial activity of antibiotics [31,32], until now it has not been applied to gonorrhea for optimizing dosage and correlating exposure with clinical outcomes [33]. Therefore, by using Monte Carlo simulation, we evaluated the probability of PK/PD target attainment by MIC (PTA), as well as taking into account the MIC distribution (CFR), which is indicative of treatment success. 

At EUCAST susceptibility breakpoints, all antibiotics except for azithromycin and the lowest dose of ceftriaxone provided high (>90%) probability of treatment success (Figure 2). Moreover, the highest doses of ceftriaxone also showed adequate results for higher MICs (1 g IM would cover an MIC of 0.25 mg/L, and 2 g/day IV would cover an MIC of 1 mg/L). Regarding azithromycin in monotherapy, 1 g would be an option only when the MIC is 0.064 mg/L or lower, and 2 g if the MIC is equal to or lower than 0.125 mg/L. 

Empirical treatments with antimicrobial drugs (i.e., without susceptibility testing) provide progressive development of decreasing susceptibility, which threatens personal and public health. The estimation of CFR is a very useful tool to guide empirical treatments, since it allows us to know which antimicrobial and dose regimen would have the best likelihood of success to treat bacterial isolates from a particular hospital or geographical area. Considering the MIC distribution of our isolates, CFR values—indicative of the probability of empirical treatment success (Table 4)—allow us to confirm that ceftriaxone (even at the lowest dose: 250 mg) and oral cefixime are good candidates to empirically treat gonorrhea (CFR around 100%) in our geographical area. On the other hand, ciprofloxacin should not be used empirically. For both ESCs and ciprofloxacin, the probability of treatment success was correlated with the susceptibility rate. However, the susceptibility rate of azithromycin was much higher than the estimated CFR. This discrepancy may be explained in part by the fact that, in contrast to β-lactams—which do not accumulate within the cell (extracelluar: intracellular ratio of 1)—azithromycin achieves intracellular concentrations several-fold higher than in plasma and extracellular fluid [34]. Taking into account that *N. gonorrhoeae* presents intracellular localization, the treatment success of azithromycin may be underestimated.

Our results are consistent with the European guidelines for the diagnosis and treatment of gonorrhea in adults [7], and with the CDC [8], both of which recommend ceftriaxone monotherapy for the treatment of uncomplicated gonorrhoeae. Because *N. gonorrhoeae* remains highly susceptible to ceftriaxone, and azithromycin resistance is increasing, prudent use of antimicrobial agents supports limiting their use. In spite of that, continuous monitoring for the emergence of resistance to this cephalosporin is necessary in order to ensure the efficacy of the recommended regimens. In this sense, surveillance programs and health care provider reports of treatment failures are essential.

For empirical treatment, the WHO recommends that the selected gonorrhea treatment should have ≥95% probability of being effective—that is, 5% or fewer of gonococcal isolates are likely to be resistant to the antimicrobial used for first-line empirical treatment [35]. In our opinion, PK/PD analysis must be also considered; in this sense, the selection of the most adequate antibiotic and dosing regimen should be based on local epidemiology. Moreover, PK/PD analysis results are also beneficial when the MIC value is known, and in the case of ceftriaxone, would allow the selection of lower doses. In previous studies, PK/PD analysis has been shown to be a useful tool for the surveillance of antimicrobial activity, as a complement to the simple assessment of MIC values [32,36,37,38]. Thus, it could be applied to guide therapy along with surveillance programs of gonococcal resistance. In this sense, the surveillance of gonococcal antimicrobial resistance could be implemented with PK/PD analysis by using the susceptibility data collected by the Euro-GASP. 

This study presents some limitations. First, PK/PD analysis was performed by using the mean and standard deviation of the PK parameters, without considering the potential influence of covariates on the PK profile; second, our simulations are based on plasma concentrations and, therefore, dosing recommendations may be more beneficial for gonococcal bacteremia than for extragenital infection. 

In spite of these limitations, our study represents a good example of how to combine molecular epidemiology, antimicrobial surveillance, and PK/PD analysis to provide quality data to support treatment guidelines—especially empirical treatments. 

## 5. Conclusions

In our geographical area, multiple distinct populations of *N. gonorrhoeae* were identified. We can confirm that ceftriaxone (even at the lowest dose: 250 mg) and oral cefixime are good candidates to treat gonorrhea. For patients allergic to β-lactam antibiotics, or with pharyngeal infections, ciprofloxacin should be only used if the MIC is known and is equal to or lower than 0.125 mg/L; this antimicrobial is not recommended to be used empirically. 

## Figures and Tables

**Table 1:** Dose regimen, PK/PD index, and pharmacokinetic parameters used for simulations.

	Ciprofloxacin	Azithromycin	Ceftriaxone	Cefixime
Dose regimen	500 mg single dose, PO	1 g, 2 g single dose, PO	0.25, 0.5, 1 g single dose, IM 2 g/day, IV	400 mg/day, PO
PK/PD index	AUC_0–∞_/MIC ≥125	AUC_0–∞_/MIC ≥59.5	*f*T_>MIC_ ≥ 20 h (IM) *f*T_>MIC_ ≥ 60% (IV)	*f*T_>MIC_ ≥ 60%
AUC_0–∞_ (mg h/L)	10.7 ± 2.6			
CL/F (L/h)		144 ± 39.5		
Ke (h^−1^)			0.082 ± 0.029	0.204 ± 0.02
Vd (L)			14.70 ± 4.93	19 ± 0.03
Fu			0.05	0.35
F			1	0.42 ± 0.045
Ka (h^−1^)			1	0.55
References	[15,16]	[17,18]	[19]	[19]

IM: intramuscular; IV: intravenous; PO: oral; AUC_0–∞_: area under the plasma concentration vs. time curve from 0 to infinity; CL: clearance; F: bioavailability; Fu: unbound fraction; Ka: absorption constant rate; Ke: elimination constant rate; Vd: volume of distribution.

**Table 2:** Antimicrobial susceptibility of 202 *N. gonorrhoeae* isolates collected in 2017–2019 (*n* = 202).

Antimicrobial	MIC Range (mg/L)	MIC_50_ (mg/L)	MIC_90_ (mg/L)	R (%) EUCAST	R (%) CLSI
Penicillin	0.002–64	0.19	3	12.6	12.6
Cefixime	0.008–0.75	0.008	0.047	6.4	1.6
Ceftriaxone	0.002–0.5	0.008	0.094	2.0	0.5
Azithromycin	0.016–16	0.19	0.75	5.4 ^a^	5.4
Ciprofloxacin	0.015–64	0.012	8	48.2	45.2
Tetracycline	0.9->256	1	32	34.2	34.2

MIC: minimum inhibitory concentration; MIC50 and MIC90: minimum inhibitory concentration at which 50% and 90% of the isolates were inhibited, respectively; R: percentage of resistant isolates. ^a^: According to the epidemiological cutoff (ECOFF) value.

**Table 3:** Genotypes, MICs, and antimicrobial resistance determinants for the 35 *Neisseria gonorrhoeae* isolates sequenced by WGS.

		Genotype		Susceptibility (mg/L)							Chromosomal Mutations								
Isolate	Year	MLST	NG-MAST	P	CRO	CFM	TET	CIP	AZM	Adquired Gene	PBP2	PBP1	PorB	GyrA	ParC	S10	mtrR Promoter ^a^	MtrR	23S rRNA
2	2017	10,314	12,547	0.25	0.016	nd	0.5	3	0.125	-	Type V non-mosaic	L421P	-	S91F, D95A	S87R	V57M	-	A39T	-
3	2017	1588	3750	0.38	0.023	nd	64	8	0.125	*tet(M)*	Type XIX non-mosaic	L421P	G120K A121G	S91F, D95A	S87R	V57M	-	A39T	-
6	2017	1596	19,728	0.125	0.008	nd	0.125	0.006	0.25	-	Type 81 semi Mosaic	-	-	-	-	-	-	-	-
9	2017	1583	19,729	0.125	0.006	nd	0.25	0.004	0.125	-	Type II non-mosaic	L421P	-	-	-	-	-35A Del	-	-
11	2017	14,274	587	0.19	0.008	nd	0.5	0.006	0.75	-	Type II non-mosaic	-	-	-	-	V57M	G	-	-
17	2017	1587	13,971	>32	0.012	nd	24	1.5	0.094	*bla*_TEM-1B_, *tet(M)*	Type II non-mosaic	-	-	S91F, D95A	D86N	V57M	-	-	-
19	2017	10,935	15,728	2	0.006	nd	24	0.003	0.032	*bla*_TEM-1B_, *tet(M)*	Type XIV non-mosaic	-	-	-	-	V57M	-	-	-
24	2017	8145	19,730	2	0.004	nd	0.5	0.004	0.125	*bla*_TEM-1B_	Type XIV non-mosaic	-	-	-	-	V57M	-	-	-
31	2017	7363	11,547	0.5	0.094	nd	1	>32	0.094	-	Type X mosaic	L421P	G120N A121G	S91F, D95N	S87R, S88P	V57M	-	-	-
33	2017	7363	13,070	0.75	0.125	nd	1	>32	0.25	-	Type X mosaic	L421P	G120N A121G	S91F, D95N	S87R, S88P	V57M	-	-	-
35	2017	8143	14,306	0.125	0.008	nd	1	8	0.125	-	Type II non-mosaic		-	S91F, D95A	S87R	V57M	-	A39T	-
50	2018	14,304	13,070	0.5	0.125	nd	1	>32	0.125	-	Type X mosaic	L421P	G120N A121G	S91F, D95N	S87R, S88P	V57M	-	-	-
51	2018	7363	9184	0.38	0.064	nd	1.5	8	0.25	-	Type IX non-mosaic	L421P	G120K A121D	S91F, D95G	E91G	V57M	-35A Del	-	-
52	2018	1901	1407	0.75	0.25	nd	3	>32	0.5	-	Type XXXIV mosaic	L421P	G120K A121N	S91F, D95G	S87R	V57M	-35A Del	-	-
56	2018	10,890	1407	0.5	0.125	nd	4	>32	0.5	-	Type XXXIV mosaic	L421P	G120K, A121N	S91F, D95G	S87R	V57M	-35A Del	-	-
58	2018	1901	19,111	0.5	0.125	nd	2	>32	0.5	-	Type XXXIV mosaic	L421P	G120K, A121N	S91F, D95G	S87R	V57M	-35A Del	-	-
59	2018	1583	217	>32	0.006	nd	64	16	0.064	*bla*_TEM-1B_, *tet(M)*	Type II non-mosaic	-	G120K, A121D	S91F, D95G	D86N	V57M	-	G45D	-
61	2018	7363	15,198	0.25	0.064	nd	2	8	0.25	-	Type IX non-mosaic	L421P	-	S91F, D95G	E91G	V57M	-35A Del	-	-
7244	2018	11428	2992	0.025	0.008	<0.016	0.25	0.016	0.75	-	Type II non-mosaic	-	-	-	-	V57M	-	A39T	-
8661	2018	15,573	17,371	0.125	0.012	0.016	2	0.016	1	-	Type II non-mosaic	-	G120K, A121N	-	-	V57M	G	-	-
293	2019	9363	6765	0.19	0.012	<0.016	2	0.012	1	-	Type II non-mosaic	-	G120K, A121N	-	-	V57M	G	-	-
1941	2019	1580	470	0.125	0.03	0.016	2	0.002	1.5	-	Type 93 semi Mosaic	-	A121S	-	-	V57M	-	G45D	C2599T
1943	2019	1580	470	0.125	0.008	<0.016	2	0.002	1.5	-	Type 93 semi Mosaic	-	A121S	-	-	V57M	-	G45D	C2599T
3023	2019	9363	6765	0.25	0.016	<0.016	1.5	0.008	2	-	Type II non-mosaic	-	G120K, A121N	-	-	V57M	G	-	-
3526	2019	9363	6765	0.19	0.023	<0.016	3	0.008	0.75	-	Type II non-mosaic	-	G120K, A121N	-	-	V57M	G	-	-
3569	2019	9363	6765	0.38	0.012	<0.016	2	0.016	2	-	Type II non-mosaic	-	G120K, A121N	-	-	V57M	G	-	-
3700	2019	11,706	17,972	0.19	0.003	<0.016	1.5	2	0.75	-	Type V non-mosaic	L421P	-	S91FD95A	S87R	V57M	-	A39T	-
4458	2019	13,292	9208	0.094	0.004	nd	2	0.003	0.75	-	Type II non-mosaic	-	-	-	-	V57M	-	A39T	C2611T
4515	2019	9363	6765	0.38	0.016	0.032	1	0.008	1.5	-	Type II non-mosaic	-	G120K, A121N	-	-	V57M	G	-	-
4726	2019	9363	6765	0.19	0.016	<0.016	3	0.008	1	-	Type II non-mosaic	-	G120K, A121N	-	-	V57M	G	-	-
5903	2019	7822	14,994	0.25	0.023	<0.016	1	4	1	-	Type V non-mosaic	L421P	-	S91F, D95A	S87R	V57M	G	A39T	-
7079	2019	9363	6765	0.125	0.008	<0.016	1.5	0.012	1.5	-	Type II non-mosaic	-	G120K, A121N	-	-	V57M	G	-	-
7181	2019	9363	19,731	0.19	0.016	<0.016	3	0.004	1.5	-	Type II non-mosaic	-	G120K, A121N	-	-	V57M	G	-	-
7789	2019	7822	14,994	0.125	0.012	<0.016	0.75	2	1	-	Type V non-mosaic	L421P		S91F, D95A	S87R	V57M	G	A39T	-
9051	2019	7822	14,994	0.38	0.016	<0.016	1.5	6	1.5	-	Type V non-mosaic	L421P	-	S91F, D95A	S87R	V57M	G	A39T	-

P: penicillin; CRO: ceftriaxone; CFM: cefixime; TET: tetracycline; CIP: ciprofloxacin; AZ: azithromycin; nd: no data available; -: wild type. ^a^: G, deletion of one G (guanine), 20 nucleotides downstream of where the -35A deletion occurs.

**Table 4:** Cumulative fraction of response (CFR) calculated for all antibiotics and dosing regimens.

Antibiotic	Dosing Regimen	CFR (%)
Ciprofloxacin	500 mg, single dose, PO	51
Azithromycin	1 g single dose, PO	33
	2 g single dose, PO	64
Ceftriaxone	1 g, single dose, IM	100
	500 mg, single dose, IM	98
	250 mg, single dose, IM	96
	2 g/day, IV	100
Cefixime	400 mg/day, PO	97

PO: oral administration; IM: intramuscular; IV: intravenous.

## Data Availability

The data presented in this study are available in Supplementary Materials.

## Supplementary Materials

The following are available online at https://www.mdpi.com/article/10.3390/pharmaceutics13101699/s1, Figure S1: MIC frequency of all antimicrobials studie. Figure S2: Alignment of partial sequences of the *mtrR* promoter from *Neisseria gonorrhoeae* FA1090 (GenBank accession number NC_002946), *Neisseria gonorrhoeae* 38197 (GenBank accession number KT954125), and some *N. gonorrhoeae* isolates from this study. The -10 and -35 promoter sequences for the *mtrR* gene are shown in overlines. The 13-bp inverted repeat (IR) is underlined. Lowercase letters represent the single-base-pair deletion (-35A Del) within the IR. *—location of the single base-pair deletion (G) 20 nucleotides downstream of where the -35A deletion occurs; Table S1: Dose regimen, PK/PD index, and pharmacokinetic parameters used for simulations; Table S2: Probability of target attainment (PTA) and cumulative fraction of response (CFR) of ciprofloxacin, 500 mg, single dose, oral administration; Table S3: Probability of target attainment (PTA) and cumulative fraction of response (CFR) of azithromycin, 1 g, single dose, oral administration; Table S4: Probability of target attainment (PTA) and cumulative fraction of response (CFR) of azithromycin, 2 g, single dose, oral administration; Table S5: Probability of target attainment (PTA) and cumulative fraction of response (CFR) of ceftriaxone, 250 mg, single dose, intramuscular administration; Table S6: Probability of target attainment (PTA) and cumulative fraction of response (CFR) of ceftriaxone, 500 mg, single dose, intramuscular administration; Table S7: Probability of target attainment (PTA) and cumulative fraction of response (CFR) of ceftriaxone, 1 g, single dose, intramuscular administration; Table S8: Probability of target attainment (PTA) and cumulative fraction of response (CFR) of ceftriaxone, 2 g/day, intravenous administration; Table S9: Probability of target attainment (PTA) and cumulative fraction of response (CFR) of cefixime, 400 mg/day, oral administration.
