# Supplementary material for: Molecular Epidemiology, Antimicrobial Surveillance, and PK/PD Analysis to Guide the Treatment of Neisseria gonorrhoeae Infections
Source: Pharmaceutics. 2021 Oct 15;13(10):1699. doi: 10.3390/pharmaceutics13101699 (PMC8541456; doi:10.3390/pharmaceutics13101699)
Supplement: Supplementary file 1 [file pharmaceutics-13-01699-s001.zip › supp-conversion.pdf]

pharmaceutics

Supplementary Materials: Molecular Epidemiology, Antimicrobial Surveillance, and PK/PD Analysis to Guide the Treatment of *Neisseria gonorrhoeae* Infections

Rodrigo Alonso, Ainara Rodríguez-Achaerandio, Amaia Aguirre-Quiñonero, Aitor Artetxe, Ilargi Martínez-Ballesteros, Alicia Rodríguez-Gascón, Javier Garaizar and Andrés Canut

Pharmacokinetic/Pharmacodynamic indexes estimation

Five thousand-subject Monte Carlo simulations with Oracle®Crystal Ball Fusion Edition v.11.1.2.3.500 (Oracle USA Inc., Redwood City, CA, USA) was used to estimate the PK/PD indexes, indicative of the probability of target success. The output consisted of a probability distribution and specific confidence intervals (CIs) over particular $fT_{>MIC}$ or AUC/MIC, and MIC values, according to the European Committee on Antimicrobial Susceptibility Testing (EUCAST) approach [1]. Moreover, the PTA, defined as the probability that a specific value of a PK/PD index associated with the efficacy of the antimicrobial treatment is achieved at a certain MIC [2] was estimated. The CFR, understood as the expected population PTA for a specific antimicrobial dose and a specific population of microorganisms, was also calculated. It allowed for an estimation of the proportion of the population achieving a certain PK/PD value, given the Monte Carlo simulation and the MIC distribution of the target microorganism [2].

Table S1 shows the PK/PD index and the pharmacokinetic parameters used for simulations. Logarithmic transformation was applied to the mean and standard deviation (S.D.) of the pharmacokinetic parameters to normalize their distributions.

Table S1. Dose regimen, PK/PD index and pharmacokinetic parameters used for simulations.

	Ciprofloxacin	Azithromycin	Ceftriaxone	Cefixime
Dose regimen	500 mg single dose, PO	1 g, 2 g single dose, PO	0.25, 0.5, 1 g single dose, IM 2 g/day, IV	400 mg/day, PO
PK/PD index	$AUC_{0-\infty}$/CMI: 125	$AUC_{0-\infty}$/CMI: 59.5	$fT_{>CMI}$> 20 h (IM) $fT_{>CMI}$> 60% (IV)	$fT_{>CMI}$> 60%
$AUC_{0-\infty}$ (mg h/L)	10.7±2.6			
CL/F (L/h)		144±39.5		
Ke (h^{-1})			0.082±0.029	0.204±0.02
Vd (L)			14.70±4.9[3]	19±0.03
Fu			0.05	0.35
F			1	0.42±0.045
Ka (h^{-1})			1	0.55
References	[3,4]	[5,6]	[7]	[7]

IM: intramuscular; IV: intravenous; PO: oral; $AUC_{0-\infty}$: area under the plasma concentration *vs* time curve in a period of 24 h; CL: clearance; F: bioavailability; Fu: unbound fraction; Ka: absorption constant rate; Ke: elimination constant rate; Vd: volume of distribution.

For ciprofloxacin, $AUC_{0-\infty}$/MIC was calculated by using directly the value of $AUC_{0-\infty}$ published previously, obtained in healthy subjects who received 500 mg of ciprofloxacin as a single oral dose [8].

For azithromycin, the $AUC_{0-\infty}$/MIC was calculated as follow:

$$^{AUC_{24}}/_{\mathrm{MIC}} = {}^{D}/_{CL \cdot MIC}$$

Where AUC0-⊚ is the area under the curve plasma concentration-time from 0 to infinity (mg h/L), D is the daily dose (mg), and CL the plasma clearance (L/h) [9,10]

For intramuscular administration of ceftriaxone [9], fT>MIC (h) was calculated by using the following equations:

$$conc = \frac{F \cdot D \cdot K_a \cdot f_u}{V_d \cdot (K_a - K)} \cdot \left(e^{-K \cdot t} - e^{-K_a \cdot t}\right)$$

where F is the drug bioavailability, D is the dose, fu is the unbound drug fraction, Ka is the absorption rate constant, Vd is the distribution volume, K is the elimination rate constant.

$$fT_{>MIC}\ (h) = t_2 - t_1$$

where t1 and t2 corresponds to the time at which the drug concentration reaches the MIC in the ascendant and in the elimination phase of the plasma concentration-time curve, respectively [10].

For endovenous administration, the following equation was used [11]:

$$fT_{>MIC}\ (\%) = Ln\left(\frac{D \cdot f_u}{V_d \cdot MIC} \cdot \frac{1 - e^{-n \cdot K \cdot \tau}}{1 - e^{-K \cdot \tau}}\right) \cdot K_e \cdot 100/\tau$$

where fT>MIC (%) is the proportion of time that the free drug concentration remains above the MIC at steady-state (%), Ln is the natural logarithm, D is the dose of antibiotic administered (mg), fu is the unbound fraction, Vd is the distribution volume, K is the elimination rate constant, n is the number of administered doses that ensures that the steady state is reached (10 doses was always selected), and ⊚ is the dosing interval.

For cefixime [6], the %fT> MIC values were calculated by using the following equations:

$$conc = \frac{F \cdot D \cdot K_a \cdot f_u}{V_d \cdot (K_a - K)} \cdot \left[\left(\frac{1 - e^{-n \cdot K \cdot \tau}}{1 - e^{-K \cdot \tau}}\right) \cdot e^{-K \cdot t} - \left(\frac{1 - e^{-n \cdot K_a \cdot \tau}}{1 - e^{-K_a \cdot \tau}}\right) \cdot e^{-K_a \cdot t}\right]$$

where F is the drug bioavailability, D is the dose, fu is the unbound drug fraction, Ka is the absorption rate constant, Vd is the distribution volume, K is the elimination rate constant, n is the number of administered doses that ensures that the steady state is reached (10 doses was always selected).

$$fT_{>MIC}\ (\%) = [(t_2 - t_1)] \times 100/\tau$$

where t1 and t2 corresponds to the time at which the drug concentration reaches the MIC in the ascendant and in the elimination phase of the plasma concentration-time curve, respectively [4].

Results

Table S2. Probability of target attainment (PTA) and cumulative fraction of response (CFR) of ciprofloxacin, 500 mg, single dose, oral administration.

		AUC$_{0-\infty}$/MIC			
**CMI (mg/L)**	**PTA (%) (AUC$_{0-\infty}$/MIC>125)**	**Mean**	**Median**	**92.5%CI**	**97.5%CI**
0.0015	100	7,135	6,940	4,707	10,224
0.002	100	5,351	5,205	3,530	7,668
0.003	100	3,567	3,470	2,354	5,112
0.004	100	2,676	2,602	1,765	3,834
0.006	100	1,784	1,735	1,177	2,556
0.008	100	1,338	1,301	883	1,917
0.012	100	891	864	585	1,293
0.016	100	668	648	438	970
0.023	100	465	451	305	674
0.125	100	86	83	56	124
0.16	4	67	65	44	97
0.5	0	21	21	14	31
0.75	0	14	14	9	20
1	0	11	10	7	15
1.5	0	7	7	5	10
2	0	5	5	4	8
3	0	4	3	2	5
4	0	3	3	2	4
6	0	2	2	1	3
8	0	1	1	1	2
12	0	1	1	1	1
16	0	1	1	0	1
33	0	0	0	0	0
64	0	0	0	0	0
CFR (%)	51	1,708	820	0	

PTA: probability of target attainment; CFR: cumulative of fraction of response (population PTA for the MIC distribution).

Table S3. Probability of target attainment (PTA) and cumulative fraction of response (CFR) of azithromycin, 1 g, single dose, oral administration.

		AUC0-∞/MIC			
**CMI (mg/L)**	**PTA (%) (AUC0-∞/MIC>59.5)**	**Mean**	**Median**	**92.5%CI**	**97.5%CI**
0.016	100	520	450	254	991
0.023	100	362	313	177	689
0.032	100	260	225	127	496
0.047	100	177	153	86	337
0.064	97	130	113	63	248
0.094	75	88	77	43	169
0.125	47	67	58	32	127
0.19	16	44	38	21	83
0.25	6	33	29	16	63
0.38	1	22	19	11	42
0.5	1	17	14	8	32
0.75	0	11	10	5	21
1	0	8	7	4	16
2	0	4	4	2	8
CFR	33	196	41	63	6

PTA: probability of target attainment; CFR: cumulative of fraction of response (population PTA for the MIC distribution).

Table S4. Probability of target attainment (PTA) and cumulative fraction of response (CFR) of azithromycin, 2 g, single dose, oral administration.

		AUC$_{0\text{-}\infty}$/MIC			
**CMI (mg/L)**	**PTA (%) (AUC$_{0\text{-}\infty}$/MIC>59.5)**	**Mean**	**Median**	**92.5%CI**	**97.5%CI**
0.016	100	100	1041	514	911
0.023	100	100	724	357	634
0.032	100	100	521	257	455
0.047	100	100	355	175	310
0.064	100	100	260	128	228
0.094	100	100	177	87	155
0.125	98	100	133	66	117
0.19	74	100	88	43	77
0.25	47	100	67	33	58
0.38	16	88	44	22	38
0.5	7	66	33	16	29
0.75	1	28	22	11	19
1	0	12	17	8	15
2	0	1	8	4	7
CFR	64	137	84	421	11

PTA: probability of target attainment; CFR: cumulative of fraction of response (population PTA for the MIC distribution).

Table S5. Probability of target attainment (PTA) and cumulative fraction of response (CFR) of ceftriaxone, 250 mg, single dose, intramuscular administration.

		%*f*T (h)			
**CMI (mg/L)**	**PTA (%) (%*f*T > 20 h)**	**Mean**	**Median**	**92.5%CI**	**97.5%CI**
0.003	100	74	75	42	96
0.004	100	72	73	40	96
0.006	100	68	66	37	96
0.008	100	64	63	35	96
0.012	100	60	57	32	96
0.016	100	56	53	30	95
0.023	99	51	49	27	87
0.032	99	47	44	25	80
0.047	97	42	39	22	71
0.064	94	38	35	20	64
0.094	87	32	30	17	55
0.125	77	28	26	14	49
0.250	35	18	17	8	34
0.5	3	8	7	0	19
1	0	1	0	0	0
2	0	0	0	0	0
CFR	96	56	55	20	96

PTA: probability of target attainment; CFR: cumulative of fraction of response (population PTA for the MIC distribution).

Table S6. Probability of target attainment (PTA) and cumulative fraction of response (CFR) of ceftriaxone, 500 mg, single dose, intramuscular administration.

		**%*f*T (h)**			
**CMI (mg/L)**	**PTA (%) (%*f*T > 20 h)**	**Mean**	**Median**	**92.5%CI**	**97.5%CI**
0.003	100	79	83	48	96
0.004	100	77	79	44	96
0.006	100	74	74	43	96
0.008	100	71	70	40	96
0.012	100	67	65	37	96
0.016	100	64	61	35	96
0.023	100	60	57	32	96
0.032	100	56	52	30	95
0.047	100	51	48	27	86
0.064	99	47	44	25	79
0.094	97	41	39	22	71
0.125	94	38	35	20	64
0.250	76	28	26	14	49
0.5	34	18	17	8	34
1	4	8	7	0	19
2	0	1	0	0	6
CFR	98	64	63	27	96

PTA: probability of target attainment; CFR: cumulative of fraction of response (population PTA for the MIC distribution).

Table S7. Probability of target attainment (PTA) and cumulative fraction of response (CFR) of ceftriaxone, 1 g, single dose, intramuscular administration.

		%*f*T (h)			
**CMI (mg/L)**	**PTA (%) (%*f*T > 20 h)**	**Mean**	**Median**	**92.5%CI**	**97.5%CI**
0.003	100	84	92	52	96
0.004	100	82	89	50	96
0.006	100	79	83	47	96
0.008	100	77	80	45	96
0.012	100	74	75	43	96
0.016	100	71	71	40	96
0.023	100	67	66	37	96
0.032	100	64	62	35	96
0.047	100	59	57	32	96
0.064	100	55	53	30	94
0.094	99	50	48	27	85
0.125	99	47	44	25	79
0.250	94	37	35	19	63
0.5	75	28	26	14	48
1	34	18	17	8	32
2	4	8	7	0	19
CFR	100	71	70	32	96

PTA: probability of target attainment; CFR: cumulative of fraction of response (population PTA for the MIC distribution).

Table S8. Probability of target attainment (PTA) and cumulative fraction of response (CFR) of ceftriaxone, 2 g/day, intravenous administration.

		%$fT_{>MIC}$			
**CMI (mg/L)**	**PTA (%) ($fT_{>MIC}$ > 60%)**	**Mean**	**Median**	**92.5%CI**	**97.5%CI**
0.0030	100	100	100	100	100
0.0040	100	100	100	100	100
0.006	100	100	100	100	100
0.008	100	100	100	100	100
0.012	100	100	100	100	100
0.016	100	100	100	100	100
0.023	100	100	100	100	100
0.032	100	100	100	100	100
0.047	100	100	100	100	100
0.064	100	100	100	100	100
0.094	100	100	100	100	100
0.125	100	100	100	100	100
0.25	100	99	100	100	100
0.5	99	98	100	79	100
1	94	91	100	57	100
2	70	75	77	34	100
4	27	45	40	9	100
8	3	12	4	0	48
16	0	0	0	0	0
CFR	100	100	100	100	100

PTA: probability of target attainment; CFR: cumulative of fraction of response (population PTA for the MIC distribution).

Table S9. Probability of target attainment (PTA) and cumulative fraction of response (CFR) of cefixime, 400 mg/day, oral administration.

		%fT$_{> MIC}$			
**CMI (mg/L)**	**PTA (%) (fT $_{>MIC}$ >60%)**	**Mean**	**Median**	**92.5%CI**	**97.5%CI**
0.008	100	100	100	100	100
0.016	100	100	100	100	100
0.023	100	99	100	96	100
0.032	100	98	100	90	100
0.047	100	95	97	83	100
0.064	100	90	91	78	100
0.094	100	83	83	71	98
0.125	100	77	77	66	91
0.19	90	69	68	58	81
0.25	65	63	62	53	74
0.38	14	54	53	44	64
0.75	0	38	38	29	47
1	0	30	30	20	40
CFR	97	97	100	77	100

PTA: probability of target attainment; CFR: cumulative of fraction of response (population PTA for the MIC distribution).

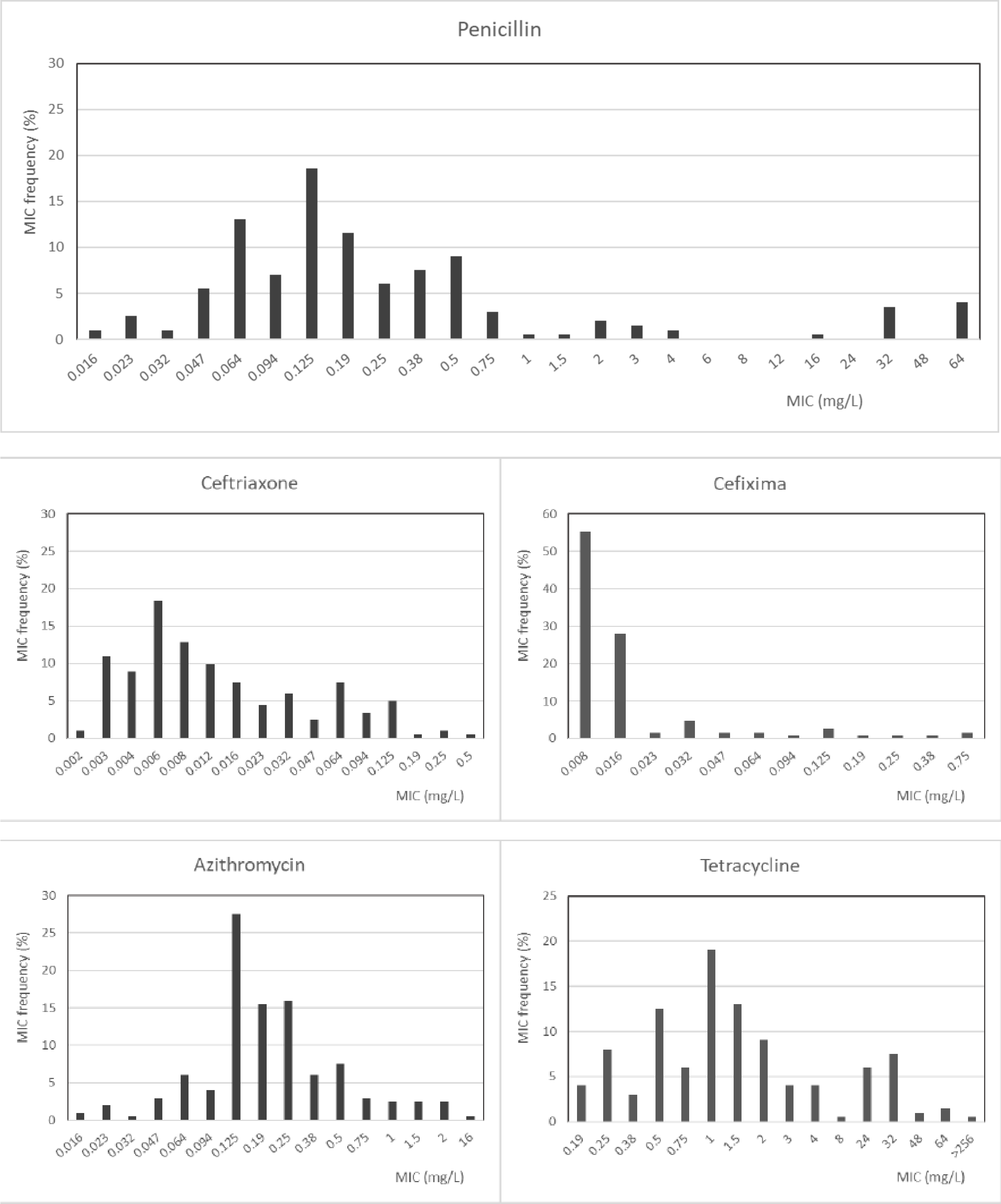
Penicillin
MIC frequency (%)
0 5 10 15 20 25 30
0.016 0.023 0.032 0.047 0.064 0.094 0.125 0.19 0.25 0.38 0.5 0.75 1 1.5 2 3 4 6 8 12 16 24 32 48 64
MIC (mg/L)
Ceftriaxone
MIC frequency (%)
0 5 10 15 20 25 30
0.002 0.003 0.004 0.006 0.008 0.012 0.016 0.023 0.032 0.047 0.064 0.094 0.125 0.19 0.25 0.5
MIC (mg/L)
Cefixima
MIC frequency (%)
0 10 20 30 40 50 60
0.008 0.016 0.023 0.032 0.047 0.064 0.094 0.125 0.19 0.25 0.38 0.75
MIC (mg/L)
Azithromycin
MIC frequency (%)
0 5 10 15 20 25 30
0.016 0.023 0.032 0.047 0.064 0.094 0.125 0.19 0.25 0.38 0.5 0.75 1 1.5 2 16
MIC (mg/L)
Tetracycline
MIC frequency (%)
0 5 10 15 20 25
0.19 0.25 0.38 0.5 0.75 1 1.5 2 3 4 8 24 32 48 64 >256
MIC (mg/L)

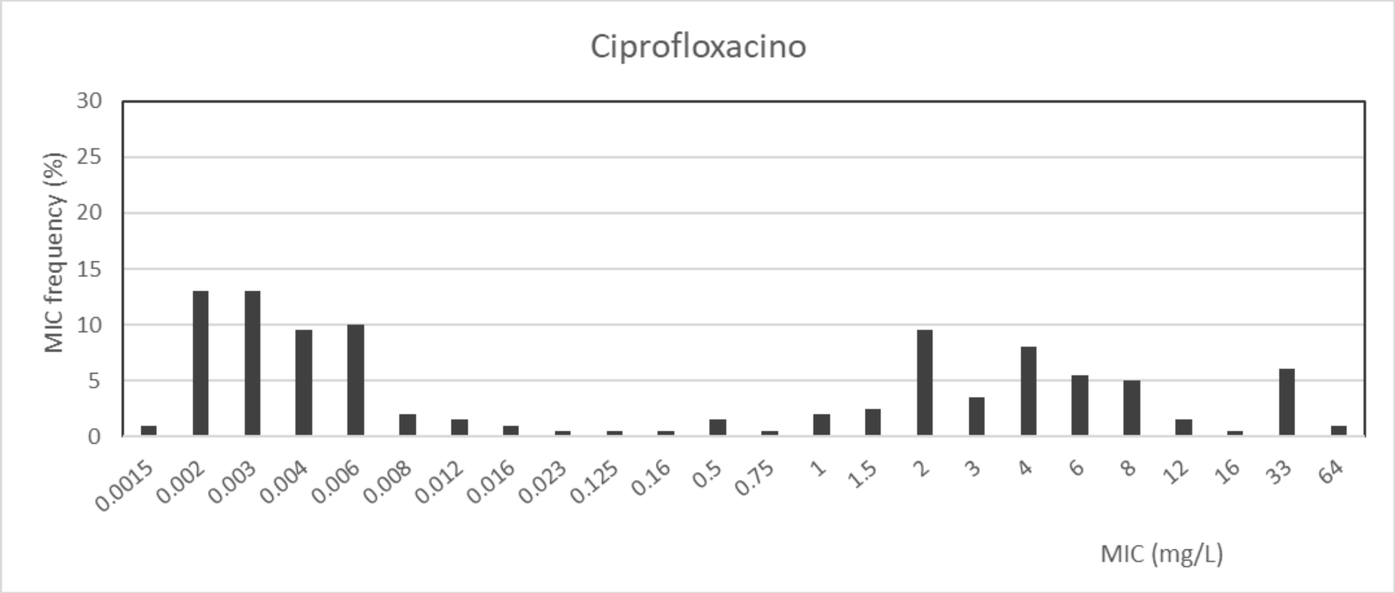

Figure S1. MIC frequency of all antimicrobials studie.

```
                   -35            -10    *                        MtrR
FA1090   ATACACGATTGCACGGATaAAAAGTCTTTTTTATAATCCGCCCTCGTCAAACCGACCCGAAACGAAAACGCCATTATGAGAAAAACCAAAACCGAAGC
1941     ATACACGATTGCACGGATaAAAAGTCTTTTTTATAATCCGCCCTCGTCAAACCGACCCGAAACGAAAACGCCATTATGAGAAAAACCAAAACCGAAGC
33       ATACACGATTGCACGGATaAAAAGTCTTTTTTATAATCCGCCCTCGTCAAACCGACCCGAAACGAAAACGCCATTATGAGAAAAACCAAAACCGAAGC
52       ATACACGATTGCACGGAT-AAAAGTCTTTTTTATAATCCGCCCTCGTCAAACCGACCCGAAACGAAAACGCCATTATGAGAAAAACCAAAACCGAAGC
9        ATACACGATTGCACGGAT-AAAAGTCTTTTTTATAATCCGCCCTCGTCAAACCGACCCGAAACGAAAACGCCATTATGAGAAAAACCAAAACCGAAGC
38197    ATACACGATTGCACGGATaCAAAGTCTTTTTTATAATCC-CAACCGTCAAACCGCGACGGAACGAAAACGCCATTATGAGAAAAACCAAAACCGAAGC
7079     ATACACGATTACACGGATaCAAAGTCTTTTTTATAATCC-CAACCGTCAAACCGCGACGGAACGAAAACGCCATTATGAGAAAAACCAAAACCGAAGC
293      ATACACGATTACACGGATaCAAAGTCTTTTTTATAATCC-CAACCGTCAAACCGCGACGGAACGAAAACGCCATTATGAGAAAAACCAAAACCGAAGC
                            IR
```

Figure S2. Alignment of partial sequences of the *mtrR* promoter from *Neisseria gonorrhoeae* FA1090 (GenBank accession number NC_002946), *Neisseria gonorrhoeae* 38197 (GenBank accession number KT954125) and some *N. gonorrhoeae* isolates from this study. The -10 and -35 promoter sequences for *mtrR* gene is shown in overlines. The 13-bp inverted repeat (IR) is underlined. Lowercase letter represents the single-base-pair deletion (-35A Del) within the IR. *, location of the single base-pair deletion (G) 20 nucleotides downstream of where the -35A deletion occurs.

References

1. Mouton JW, Brown DF, Apfalter P, Cantón R, Giske CG, Ivanova M, et al. The role of pharmacokinetics/pharmacodynamics in setting clinical MIC breakpoints: the EUCAST approach. Clin Microbiol Infect 2012;18:E37-45.
2. Mouton JW, Dudley MN, Cars O, Derendorf H, Drusano GL. Standardization of pharmacokinetic/pharmacodynamic (PK/PD) terminology for anti-infective drugs: an update. J Antimicrob Chemother 2005;55:601-7

3. Lettieri JT, Rogge MC, Kaiser L, Echols RM, Heller AH. Pharmacokinetic profiles of ciprofloxacin after single intravenous and oral doses. Antimicrob Agents Chemother. 1992;36:993-6
4. Koomanachai P, Bulik CC, Kuti JL, Nicolau DP. Pharmacodynamic modeling of intravenous antibiotics against gram-negative bacteria collected in the United States. *Clin Ther*. 2010;32(4):766-779. doi:10.1016/j.clinthera.2010.04.003
5. Dumitrescu TP, Anic-Milic T, Oreskovic K, Padovan J, Brouwer KLR, Zuo P, Schmith VD. Development of a population pharmacokinetic model to describe azithromycin whole-blood and plasma concentrations over time in healthy subjects. Antimicrob Agents Chemother. 2013;57:3194-3201. DOI: 10.1128/AAC.02430-12
6. Soda M, Ito S, Matsumaru N, et al. Evaluation of the microbiological efficacy of a single 2-gram dose of extended-release azithromycin by population pharmacokinetics and simulation in Japanese patients with gonococcal urethritis. *Antimicrob Agents Chemother*. 2017;62(1):e01409-17. doi:10.1128/AAC.01409-17
7. Chisholm SA, Mouton JW, Lewis DA, Nichols T, Ison CA, Livermore DM. Cephalosporin MIC creep among gonococci: time for a pharmacodynamic rethink? J Antimicrob Chemother. 2010;65:2141-8. doi: 10.1093/jac/dkq289.
8. Lettieri JT, Rogge MC, Kaiser L, Echols RM, Heller AH. Pharmacokinetic profiles of ciprofloxacin after single intravenous and oral doses. Antimicrobial Agents and Chemotherapy. 1992;36:993-6
9. Chisholm SA, Mouton JW, Lewis DA, Nichols T, Ison CA, Livermore DM. Cephalosporin MIC creep among gonococci: time for a pharmacodynamic rethink? Journal of Antimicrobial Chemotherapy. 2010;65:2141-8. doi: 10.1093/jac/dkq289.
10. Ibar-Bariain M, Rodríguez-Gascón A, Isla A, Solinís MA, Canut-Blasco A. Application of pharmacokinetic/pharmacodynamic analysis to evaluate the adequacy of antimicrobial therapy for pediatric acute otitis media in Spain before and after the introduction of the PCV7 vaccine. Rev Esp Quimioter. 2019;32(2):121-129
11. Asín E, Isla A, Canut A, Rodríguez Gascón A. Comparison of antimicrobial pharmacokinetic/pharmacodynamic breakpoints with EUCAST and CLSI clinical breakpoints for Gram-positive bacteria. Int J Antimicrob Agents. 2012;40(4):313-322. doi:10.1016/j.ijantimicag.2012.06.005.
